# Multiple ictal onset patterns underlie seizure generation in seizure-free patients with temporal lobe epilepsy surgery: an SEEG study

**DOI:** 10.1007/s00701-021-04960-7

**Published:** 2021-09-04

**Authors:** Cuiping Xu, Xiaohua Zhang, Xiaoming Yan, Kai Ma, Xueyuan Wang, Xi Zhang, Duanyu Ni, Liang Qiao, Tao Yu, Guojun Zhang, Yuping Wang, Yongjie Li

**Affiliations:** 1 Beijing Institute of Functional Neurosurgery, Xuanwu Hospital, Capital Medical University, No. 45 Changchun Street, Xicheng District, Beijing, 100053 China; 2 Department of Neurology, Xuanwu Hospital, Capital Medical University, Beijing, 100053 China

**Keywords:** Temporal lobe epilepsy, Stereoelectroencephalography, Ictal onset patterns, Surgical outcome

## Abstract

**Purpose:**

Seizure originates from different pathological substrate; however, the same pathologies may have distinct mechanisms underlying seizure generation. We aimed to improve the understanding of such mechanisms in patients with temporal lobe epilepsy (TLE) by investigating the stereoelectroencephalography (SEEG) ictal onset patterns (IOPs).

**Methods:**

We analyzed data from a cohort of 19 consecutive patients explored by SEEG and had 1–3-year seizure-freedom following temporal lobe resection.

**Results:**

Six IOPs were identified. They were low voltage fast activity (LVFA) (36.5%), rhythmic spikes or spike-waves at low frequency and with high amplitude (34.1%), runs of spikes (10.6%), rhythmic sharp waves (8.2%), low frequency high amplitude repetitive spiking (LFRS) (7.1%), and delta activity (3.5%). All six patterns were found in patients with mesial temporal onset and only two patterns were found in patients with temporal neocortical onset. The most prevalent patterns for patients with mesial temporal onset were rhythmic spikes or spike-waves, followed by LVFA with a mean discharge rate 74 Hz. For patients with temporal neocortical onset, the most prevalent IOP pattern was LVFA with a mean discharge rate 35 Hz, followed by runs of spikes. Compared with Lateral TLE (LTLE), the duration between the onset of the IOPs to the onset of the symptom was longer for patients with MTLE (Mesial TLE) (MTLE:55.7 ± 50.6 s vs LTLE:19.5 ± 16.4 s).

**Conclusion:**

Multiple IOPs underlie seizure generation in patients with TLE. However, the mesial and lateral temporal lobes share distinct IOPs.

## Introduction

Temporal lobe epilepsy (TLE) is the most common refractory partial epilepsy, and it is suited for surgical treatment. According to the international classification of epileptic syndromes, TLE can be distinguished to two subtypes: mesial TLE (MTLE) and lateral TLE (LTLE) [12]. About two-thirds of patients are MTLE and they have the features of hippocampal sclerosis (HS) on MRI and characteristic ictal EEG rhythm (4- to 7-Hz frequency). One-third of patients are LTLE and they have the features of no HS and < 4 Hz ictal EEG rhythm. Diagnosing TLE is difficult in view of the lack of the EEG feature, seizure semiology, and HS on MRI. So intracranial electroencephalography (iEEG) is indispensable for identifying the epileptogenic zone (EZ). The ictal iEEG showed that the fast activity (20–30 Hz) and low-frequency high-amplitude periodic spikes (LFRS) are the major patterns of hippocampus and the parahippocampal region [1, 6, 13]. However, some studies have reported alternative ictal onset patterns (IOPs) [9]. In this investigation, we sought to characterize the SEEG IOPs in seizure-free patients with TLE surgery.

## Methods

### Patient selection

Among 132 patients who underwent SEEG in our epilepsy center between January 2016 and December 2017, 30 patients underwent anterior temporal lobectomy. For patients with TLE, a clear mesial sclerosis, concordant scalp EEG, concordant neuropsychological findings, and a typical history, one stage resection is suggested, and if the anatomical findings on MRI are not clearly showing a mesial sclerosis, or if the semiology more indicates a temporal plus situation and the language dominant side, the SEEG is a priority. We included cases with good surgical outcomes (Engel classΙ) and 95% reduction in seizure frequency, and a follow-up period ranging from 1 to 3 years. After careful screening, 19 consecutive patients were included. All of the patients had a pre-operative evaluation including a detailed history and neurological examination, MRI, scalp video-EEG, and SEEG recording of seizures.

### Scalp EEG and stereoelectroencephalography recordings

According to the international 10–20 system, the scalp video-EEG was recorded (Micromed; Treviso, Italy). On the basis of non-invasive investigation and hypotheses about the localization of the EZ, multiple lead depth electrodes were placed (Talairach et al., 1974). The electrodes contain 10–16 contacts with length 2 mm, diameter 0.8 mm, and 1.5 mm apart. Then the seizures were recorded on a 128-channel Micromed system with a sampling rate 1024 Hz. A high-pass filter 0.008 Hz and low-pass filter 500 Hz were selected. These electrodes were placed in mesial and lateral temporal regions as well as extra-temporal regions in some patients. Subsequently, a post-operative computerized tomography (CT) scan was used to verify the absence of bleeding and the precise location of each electrode. Pre-operative MRI and post-operative CT fusion was performed to determine the position of each contact anatomically.

### Signal analysis

Visual analysis of the ictal recordings was carried out to identify the IOPs. Two epileptologists independently reviewed the SEEG data and the discrepancies were solved after discussion with a professional neuroelectrophysiologist. If the rhythmic discharge lasts for longer than 1.5 s, the sustained pattern is identified [5]. The seizure onset was defined as the earliest sustained rhythmic activity of SEEG discharge and subsequent appearance of the seizure symptom [7]. The IOPs were evaluated on the earliest involved electrodes. Onset morphology was described for each seizure. We visually assessed the position of each intracranial contacts basing on co-registration of pre-operative MRI and the post-operative CT.

### Surgical and post-operative follow-up

All the patients underwent standard anterior temporal lobectomy with resection of both mesial and lateral temporal cortex. The seizure outcome following the resection was classified according to Engel’s classification [4].

### Approval

The study was approved by the Ethics Committee of Xuanwu Hospital, Capital Medical University, China, according to the Declaration of Helsinki. All patients and their relatives signed informed consent.

## Results

### Clinical characteristics

Nineteen patients (male: 7; female: 12) with SEEG assessment were selected for this study. The average age at SEEG was 26.4 ± 7.6 years (range 12.0–40.0 years). The average duration of epilepsy before SEEG was 14.8 ± 7.6 years (range, 3.0–30.0 years). Five patients had a visible lesion on MRI (hippocampal sclerosis (HS): 3; abnormal signal: 2) and 14 patients had no visible lesion on MRI. All the patients were Engel class Ι.The average follow-up time was 24 ± 8 months (12–37 months). Patients’ clinical characteristics are summarized in Table 1.

**Table 1:** Patients’ clinical characteristics

Patient/gender	Age at onset (year)	Age at surgery (year)	Duration	Interictal EEG	Ictal EEG	Seizure type	MRI	MEG	PET	Surgery type	Histology	Follow-up (months)	Surgical outcome
1/f	2	27	25	B T spikes, B F and T slow waves	Rt T rhythmic slow waves	CPS	Normal	-	-	Rt T	HS and FCD	28	Ι
2/m	10	17	7	Rt T and P slow waves	Rt hemispheric rhythmic slow waves	CPS	Tumor in Rt T	Rt T	-	Rt T	GG and HS	37	Ι
3/m	21	27	6	B T spikes (predominately Lt T)	Lt T rhythmic slow waves	CPS	Normal	-	-	Lt T	Gliosis	24	Ι
4/m	12	25	13	Rt T spikes and slow waves	Electrodecremental	GTCS	Normal	Rt T and P	-	Rt T	HS and FCD	24	Ι
5/m	14	26	12	Lt T spikes	Rt T rhythmic slow waves	CPS	Normal	Rt T and I, Lt T and I	Rt T	Rt T	FCD	33	Ι
6/m	6	24	18	Lt T spikes	Motion artifacts	GTCS	Normal	-	-	Lt T	HS and FCD	12	Ι
7/f	28	38	10	Rt T slow waves	Rt T rhythmic slow waves	CPS	Normal	Rt T	-	Rt T	HS and FCD	25	Ι
8/f	21	29	8	Rt T spikes	Rt T rhythmic sharp waves	CPS	Normal	-	Rt T	Rt T	FCD	12	Ι
9/f	9	12	3	Rt T spikes, Rt F and T slow waves	Rt F and T rhythmic slow waves	CPS	Normal	-	Rt T and P	Rt T	HS	23	Ι
10/f	13	33	20	Rt T spikes	Rt T slow waves	CPS	HS in Lt T	-	Lt T	Lt T	HS and FCD	22	Ι
11/f	7	19	12	B T spikes (predominately Lt T)	Lt T rhythmic slow waves	CPS	Normal	-	-	Lt T	HS and FCD	30	Ι
12/f	12	32	20	Lt T and P slow waves	Lt T rhythmic slow waves	GTCS	HS in Lt T	-	Lt T	Lt T	HS and FCD	36	Ι
13/f	12	24	12	B T spikes, B F and T slow waves	Lt F and T rhythmic slow waves	CPS	Normal	B T	Lt T	Lt T	HS and FCD	36	Ι
14/f	20	27	7	Rt T spikes	Rt T rhythmic slow waves	CPS	HS in Rt T	Rt T and I	Lt T	Rt T	HS and FCD	20	Ι
15/f	3	23	20	B F and T spikes	Rt T, P and O fast activity	CPS	Normal	B T	Rt P, Lt F and B T	Rt T	HS and FCD	19	Ι
16/f	18	40	22	B T spikes	Rt T rhythmic slow waves	GTCS	Normal	B T	-	Rt T	FCD	27	Ι
17/m	6	32	26	Rt hemispheric spike and waves	Rt F, C and T rhythmic sharp waves	Hypermotor	Normal	-	-	Rt T	FCD	12	Ι
18/m	4	34	30	B T spikes	Rt T rhythmic slow waves	CPS	Tumor in Rt T	B T	Lt F and T	Rt T	GG	21	Ι
19/f	2	13	11	Rt F, T and P spikes	Rt F and T fast activity	Hypermotor	Normal	-	Rt T and I	Rt T	FCD	18	Ι

Abbreviations: *B*, bilateral; *C*, central; *CPS*, complex partial seizure; *F*, frontal; *FCD*, focal cortical dysplasia; *GG*, Ganglioglioma; *GTCS*, generalized tonic clonic seizure; *HS*, hippocampal sclerosis; *I*, insular; *Lt*, left; *O*, occipital; *P*, parietal; *Rt*, right; *T*, temporal

### Ictal semiology and scalp ictal EEG patterns

A total of 66 scalp EEG seizures were recorded. Forty-three (65.2%) were complex partial seizure, 14 (21.2%) were secondary generalized tonic–clonic seizure, and 9 (13.6%) were hypermotor seizure. Multiple seizures were recorded in 6 of 19 patients. The ictal EEG patterns were assessed on the basis of the first obvious ictal rhythm. A total of 66 seizures were reviewed and three IOPs were finally indentified. The most prevalent patterns were theta/alpha (5–13 Hz) (57.6%), followed by delta (2–4 Hz) (28.8%), and then beta (≧14 Hz) (3.0%). Seven (10.6%) seizures provided worthless location information.

### SEEG ictal onset patterns

**Fig. 1:**
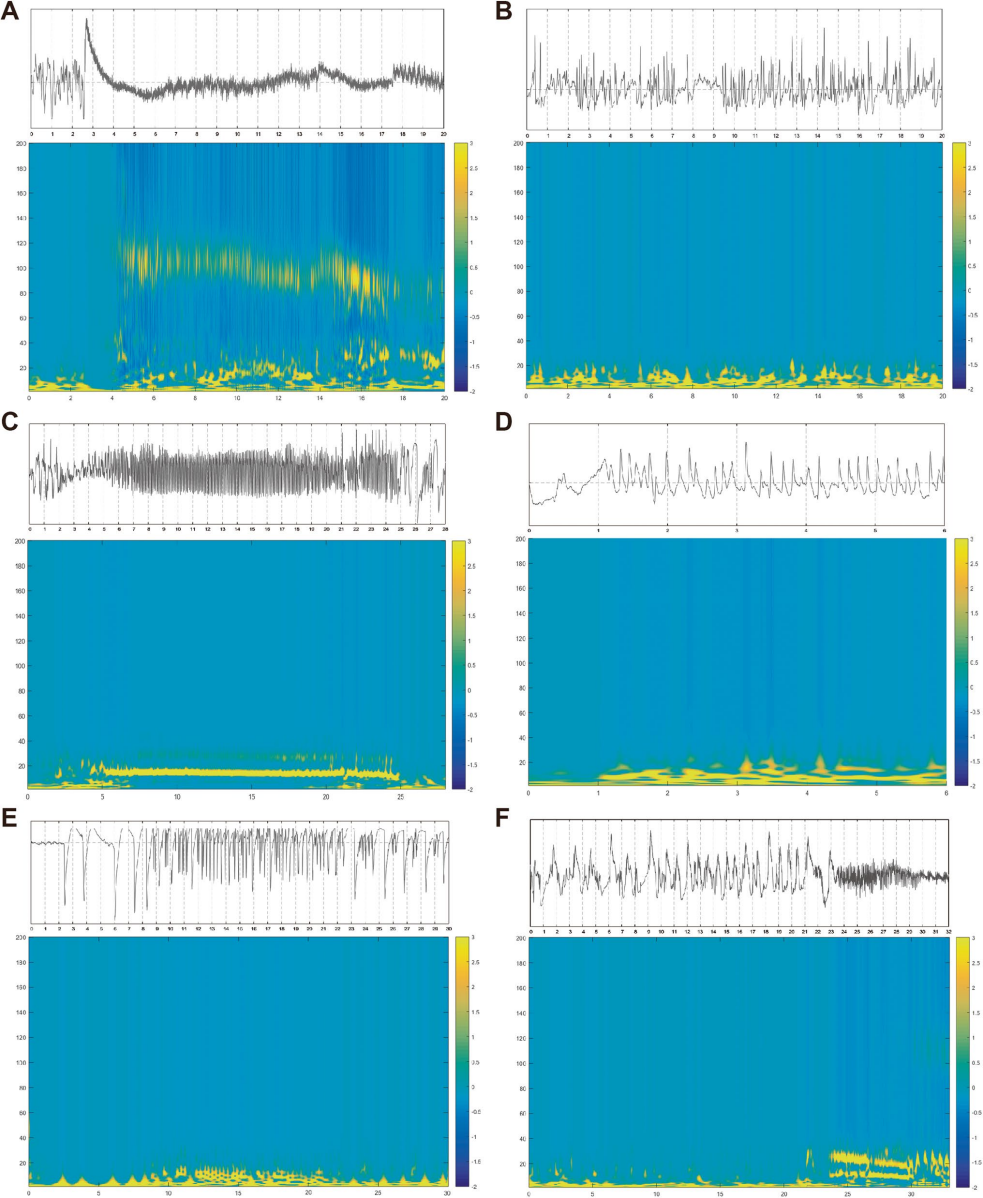
Six different IOPs according to the time–frequency analysis. **A** Low-voltage fast activity (LVFA). **B** Rhythmic spikes or spike-waves with high amplitude and low frequency. **C** Runs of spikes. **D** Rhythmic sharp wave. **E** low-frequency high-amplitude periodic spikes (LFRS). **F** Delta activity

A total of 85 seizures were reviewed (range 2–10) and six main IOPs were identified (Fig. 1). The most prevalent patterns were LVFA (36.5%). It is followed by rhythmic spikes or spike-waves with high amplitude and low frequency (34.1%), then runs of spikes (10.6%), and then rhythmic sharp wave (8.2%), LFRS (7.1%) and delta activity (3.5%). The remarkably high prevalence of patterns was LVFA (7/19 patients, 36.8%), followed by rhythmic spikes or spike-wave (5/19 patients, 26.3%), and then rhythmic sharp (3/19 patients, 15.8%). The LFRS was the fourth one (2/19 patients, 10.5%). The runs of spikes and delta activity were the last two patterns and shared the same proportion (both 1/19 patients, 5.3%).

Three patients had the features of HS on MRI and the corresponding IOPs were LVFA, rhythmic spikes or spike-waves, and delta activity, respectively. Two patients had abnormal signal on MRI, and the corresponding IOPs were LVFA and rhythmic sharp wave. For patients with MRI negative, the most prevalent patterns were LVFA and rhythmic spikes or spike-waves.

**Fig. 2:**
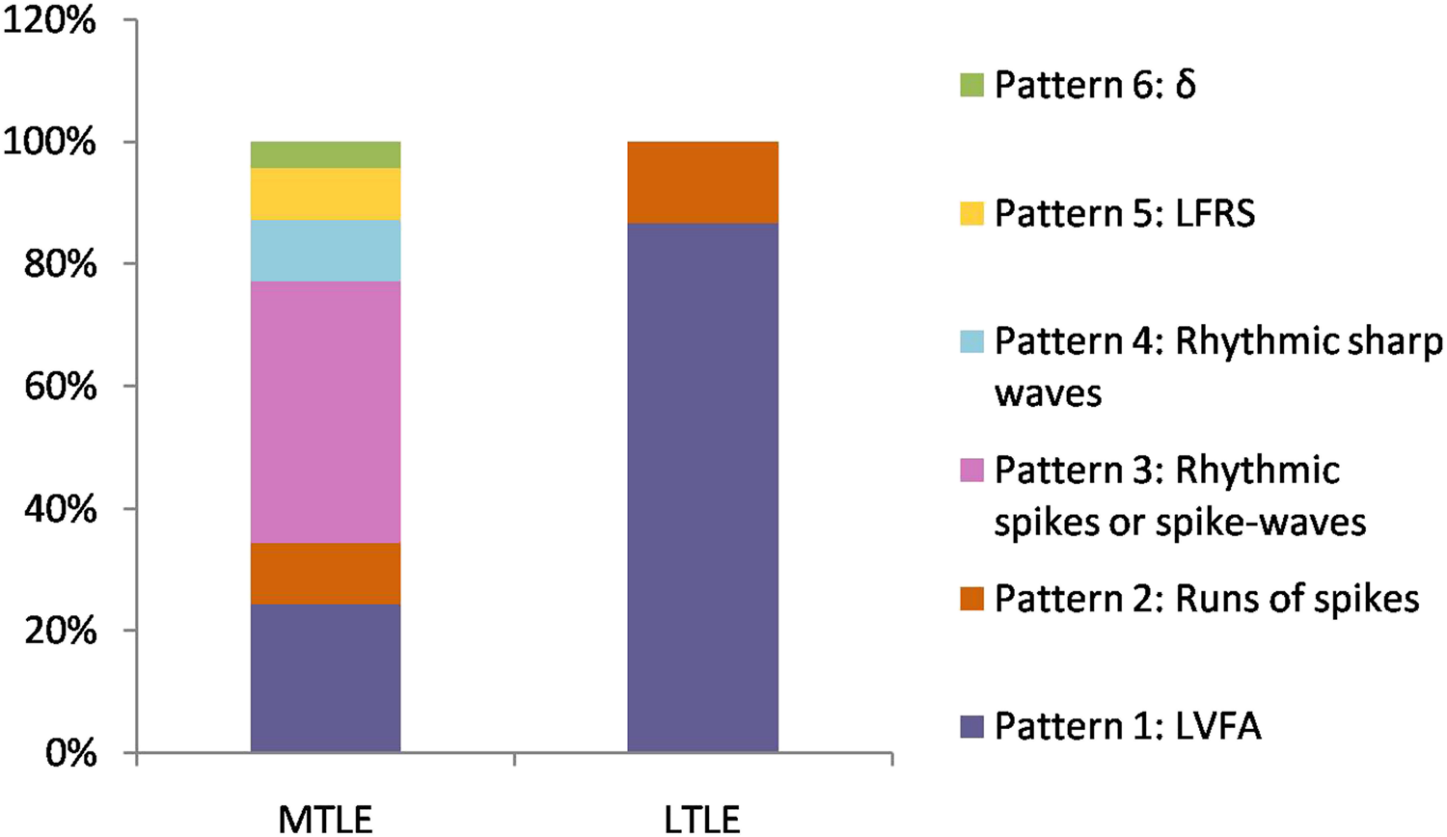
Frequency of the IOPs between MTLE and LTLE. The frequency is shown in percentage of the total seizures for the two groups

According to the site of the first sustained rhythmic activity, fifteen patients originated from the mesial temporal and four patients originated from the temporal neocortical. The IOPs varied according to the site of ictal onset (Fig. 2). The most prevalent patterns for patients with mesial temporal onset were rhythmic spikes or spike-waves, followed by LVFA with a mean discharge rate 74 Hz, then runs of spikes, rhythmic sharp wave, and then LFRS and delta activity. For patients with temporal neocortical onset, two IOPs were found. One was LVFA with a mean discharge rate 35 Hz and the other was runs of spikes. Compared with LTLE, the duration between the onset of the IOPs to the onset of the symptom was longer for patients with MTLE (MTLE: 55.7 ± 50.6 s vs LTLE: 19.5 ± 16.4 s).

### Histopathological examination

All subjects had histopathological examination. For patients with MTLE, 80% (*n* = 12) was HS and 13.3% (*n* = 2) was FCD. For patients with LTLE, 75% (*n* = 3) was FCD and 25% (*n* = 1) was ganglion glioma。

## Discussion

The major purpose of this study was to assess the SEEG IOPs in seizure-free patients following TLE surgery. In order to assess the IOPs, the group only consisted of the SEEG patients. One hundred and thirteen patients who underwent one stage anterior temporal lobe resection were excluded. Six distinct morphological patterns were identified at seizure-onset. They were LVFA, rhythmic spikes or spike-waves, runs of spikes, rhythmic sharp wave, LFRS, and finally delta activity. Notably, the IOPs varied according to the position of seizure onset. Six patterns were defined in MTLE; however, only two IOPs, LVFA, and runs of spikes were found in LTLE. The rhythmic spikes or spike-waves were the most prevalent IOP in patients with MTLE and the LVFA was the prevalent IOP in patients with LTLE. In view of favorable surgical outcome, we thought that the EZ had been removed and the seizure onset pattern was an IOP. The single spike or bursts of spikes with short duration at onset were excluded, because these patterns did not correlate with IOP evolution or seizure outcome following surgery [3].

Diagnosing MTLE is reasonable based on the three major features: presence of obvious electro-clinical characteristics, symptoms (such as a history of febrile convulsions, an ictal epigastric sensation, and oral automatisms), and HS on MRI. However, according to Wieser [15] viewpoint, diagnosing MTLE may be difficult due to the evidence is insufficient in many patients, and it is undefined that how many and which features are needed and whether HS is necessary. In this study, 12 patients had no HS on MRI. However, most of the patients obtained histopathological diagnosis of HS following surgery. So, a significant portion of the MTLE had MRI-normal hippocampus. At this point, we need to rely on the SEEG for diagnosing MTLE. On the other hand, because the seizure symptom may be the same even if one or more of these specific mesial temporal structures (the amygdalohippocampal complex, parahippocampal gyrus, and the entorhinal cortex) is the leader of the seizure [2], it is impossible to know the seizure onset zone without SEEG.

Rhythmic spikes or spike-waves were the most predominant pattern in patients with MTLE. According to Perucca’s view, this pattern is a hypersynchronous neuronal discharge due to enhanced excitation [11]. Periodic spikes are features of neuronal loss and gliosis in the hippocampus [10], whereas some studies reported that patients with amygdalar atrophy or normal temporal lobe also showed this pattern. LFRS was another periodic spike with a slower frequency, and it was the predominant IOP in MTLE. Slower LFRS indicates more severe mesial temporal sclerosis. However, in our cohort, LFRS was less common in MTLE. One reason may be that the patients in that study had a mesial temporal atrophy/sclerosis documented by MRI [11], and our patients had MRI-normal mesial temporal structures.

LVFA was the most prevalent pattern for LTLE. It has been proposed that LVFA in the beta-gamma frequency has the greatest localizing value for determining the EZ [14]. However, patients with neocortical temporal epilepsy shown a lower frequency LVFA (beta range) than that with extratemporal epilepsy (gamma range) [8]. Contrary to Wendling et al.’s review, Velasco suggested that LVFA was relevant to a more extensive EZ due to the functional decoupling with distant cortex [13]. Yet, some studies have confirmed that LVFA portends a favorable prognosis. In this study, our data is consistent with this conclusion, and histopathologic analyses demonstrated that LVFA was associated with FCD. It is worth mentioning that during visual analysis, we should elevate the sensitivities to recognize the low-voltage fast activity.

## Conclusion

Multiple IOPs underlie seizure generation in patients with TLE. However, the mesial and lateral temporal lobes share distinct IOPs.

### Limitations

There are several limitations in this study. Major limitations include the small sample size and the retrospective nature of the study. Another limitation is that we did not compare the different patterns between mesial atrophy/sclerosis and MRI-normal mesial temporal structures due to the small sample size. We will perform further investigation with this aspect.
